# Deep learning-based hologram generation using a white light source

**DOI:** 10.1038/s41598-020-65716-4

**Published:** 2020-06-02

**Authors:** Taesik Go, Sangseung Lee, Donghyun You, Sang Joon Lee

**Affiliations:** 1 Center for Biofluid and Biomimic Research, Department of Mechanical Engineering, Pohang University of Science and Technology, Pohang, 37673 Republic of Korea; 2 Flow Physics and Engineering Laboratory, Department of Mechanical Engineering, Pohang University of Science and Technology, Pohang, 37673 Republic of Korea

**Keywords:** Applied optics, Imaging and sensing, Microscopy, Interference microscopy

## Abstract

Digital holographic microscopy enables the recording of sample holograms which contain 3D volumetric information. However, additional optical elements, such as partially or fully coherent light source and a pinhole, are required to induce diffraction and interference. Here, we present a deep neural network based on generative adversarial network (GAN) to perform image transformation from a defocused bright-field (BF) image acquired from a general white light source to a holographic image. Training image pairs of 11,050 for image conversion were gathered by using a hybrid BF and hologram imaging technique. The performance of the trained network was evaluated by comparing generated and ground truth holograms of microspheres and erythrocytes distributed in 3D. Holograms generated from BF images through the trained GAN showed enhanced image contrast with 3–5 times increased signal-to-noise ratio compared to ground truth holograms and provided 3D positional information and light scattering patterns of the samples. The developed GAN-based method is a promising mean for dynamic analysis of microscale objects with providing detailed 3D positional information and monitoring biological samples precisely even though conventional BF microscopic setting is utilized.

## Introduction

Conventional bright-field (BF) microscopy using a white light source is a widespread imaging technique used in various areas, including industrial, biological, and medical fields. BF images have been commonly used to observe microscale objects, such as emulsions, microorganisms, and biological samples^1–5^. Detection or diagnostic sensitivity is not high (usually less than 90% accuracy), and 3D monitoring of samples in a large volume is especially limited because BF images only provide two-dimensional (2D) amplitude information within the shallow depth of focus (DOF).

Digital holographic microscopy (DHM) is a powerful imaging technique that encrypts the 3D information of a test sample into a single shot of 2D interference patterns (i.e., hologram). DHM has a deep observation depth that can overcome the technical limitations of BF microscopy by using a coherent light source. Therefore, DHM has been used in various fields including accurate biological sample monitoring^6–14^, environmental monitoring^15–17^, and particle or cell dynamic analysis^18–20^, because amplitude and phase information can be noninvasively obtained from the hologram without any labeling and mechanical scanning procedures. DHM systems with off-axis configuration can directly obtain phase information related to 3D morphological, mechanical, and biochemical properties^21^. However, a complicated interferometric experimental setup, such as common-path and Mach-Zhender setups, is required in such systems. Digital in-line holographic microscopy (DIHM) has a relatively simple optical setup, that is, it only requires a single beam path. DIHM has been widely used in investigating various microscale flows and 3D dynamic motions of particles or cells by extracting 3D positional information from numerically reconstructed holographic images^22–25^. Both types of DHM provide several valuable features. However, the holographic image can not be acquired with conventional BF imaging setting because partially coherent light source and a pinhole are at least required^15–17^.

Recently, deep learning has received considerable interest because of its usefulness and effectiveness in various research areas^26^. This artificial neural network is a multilayered one and has been successfully applied to various fields where numerous data are available. Previous studies on DHM were incorporated with deep learning and addressed on various fields, including the enhancement of the spatial resolution of holographic images^27,28^, improvement of hologram reconstruction^29–32^, phase retrieval^33^, compensation of phase aberration^34^, noise reduction in tomograms^35^, autofocusing and depth location prediction^36,37^, and classification and monitoring of various bio-samples^12,17,38,39^.

In the present study, a new deep learning-based framework is proposed to overcome the technical limitations of conventional BF microscopy (i.e., insufficient information about the test sample) and DHM (i.e., necessity of additional optical elements). Generative adversarial network (GAN)^31,40,41^ is applied to convert a BF image of a sample located at a certain depth into an equivalent holographic image of the sample. To the best of our knowledge, there is no previous study for converting BF images to holographic images using other approaches including machine or deep learning based methods. The hybrid BF and hologram imaging technique was combined with a microfluidic device to gather the training and test sets of polystyrene microspheres and erythrocytes located at diverse depth positions (*z*). The quality of the generated hologram transformed from the BF image via GAN was examined by comparing the generated and ground truth holograms. As a result, the trained network was formed to effectively generate holograms with reduced background noise. After the image conversion through GAN, two parameters were extracted from the generated holograms to validate how useful this methodology is. The generated holograms demonstrate that the 3D positional information and light scattering patterns of microparticles, which cannot be obtained from original BF images, can be precisely obtained. Thus, it is possible to analyze test samples in a 3D volume in more detail, even with the use of a white light source.

## Results and discussion

### Training network

**Figure 1:**
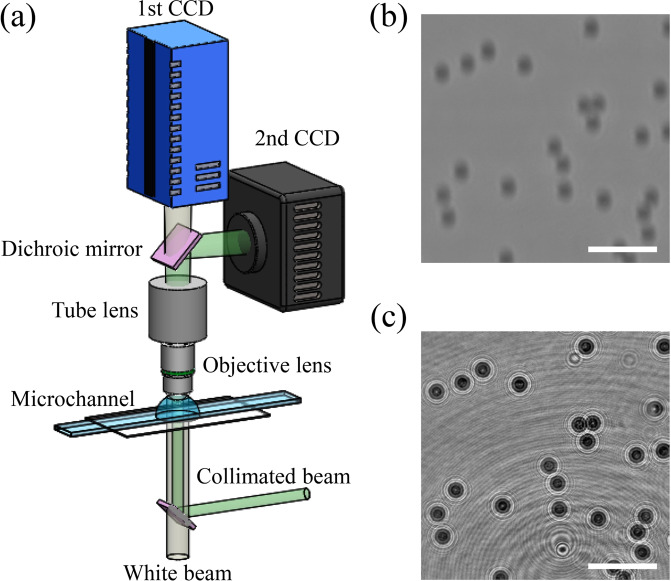
Hybrid BF and hologram imaging system. (**a**) Schematic of the experimental setup used in this study. The white light beam and green laser beam illuminated the test samples in a microchannel. The beam passing through the samples was magnified by an objective lens and tube lens. Dichroic mirror divided the beam into two separated beams. Only green light used for capturing holograms of microparticles reached the second CCD. The other light used for BF images of microparticles reached the first CCD. Defocused BF images (**b**) and corresponding holograms (**c**) of test samples were simultaneously recorded as training datasets by using the hybrid imaging system. Scale bars are 100 μm. The schematic was illustrated by the authors using SolidWorks software (Dassault Systèmes SolidWorks Corp., USA).

The hybrid imaging system was used to simultaneously record BF images and the corresponding holographic images^42^ [Fig. 1a]. The dichroic mirror only reflected green light and passed all other lights. The beam was divided into two beams for BF and hologram imaging. Only BF images were captured using the first high-speed CCD camera [Fig. 1b]. Holographic images were recorded by using the second high-speed CCD camera at the same frame rate used for recording BF images [Fig. 1c]. These two CCD cameras were connected to a delay generator to synchronizing their exposures for obtaining BF and corresponding hologram images at the same time. Suspensions of PS particles and erythrocytes were infused into a rectangular microchannel to gather numerous BF-hologram image pairs (n = 10,000 for PS particles, n = 1,050 for erythrocytes) that will be used for the training network [Fig. 1a]. The Reynolds number was maintained at 10^−2^ by adjusting the flow rate. The focal plane of the objective lens was adjusted using the *z*-axis translation stage of a microscope. Finally, lots of image pairs of microparticles located at various depth positions (*z*) were captured by using the simple microfluidic device.

**Figure 2:**
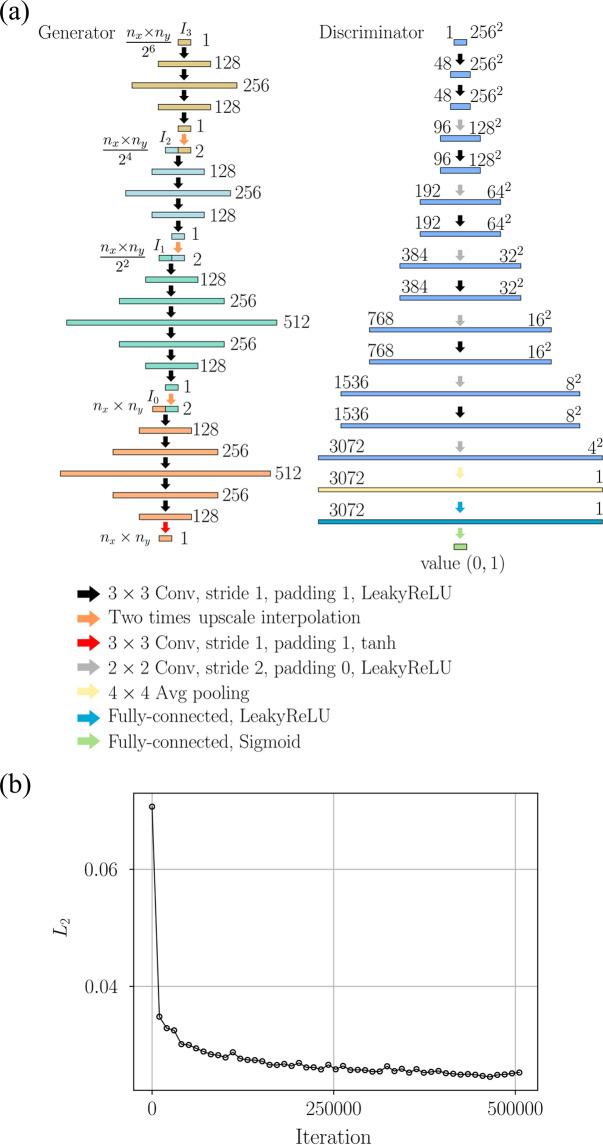
GAN structure and training process. (**a**) GAN architecture is composed of a generator and a discriminator. The numbers of feature maps or neurons are indicated on the left sides of the generator and discriminator. The sizes of feature maps are indicated on the right side of the discriminator. Different colors of arrows represent various types of connections. The merging of two blocks with different colors indicate the concatenation of feature maps. (**b**) Variation of *L*_2_ error of test data according to training iterations.

A GAN was employed to transform a BF image into a holographic image. The GAN is composed of a generator and discriminator network [Fig. 2a]. In the generator, a variation network of the original multiscale CNNs^43^ was used to transform BF images into holographic images. These multiscale CNNs can learn the structures that contain multirange spatial dependencies^44^. A variation structure of a discriminator proposed by the previous research^31^ was used for the discriminator of the present GAN. The utilized GAN was trained up to 500,000 iterations [Fig. 2b].

### Generated hologram quality

**Figure 3:**
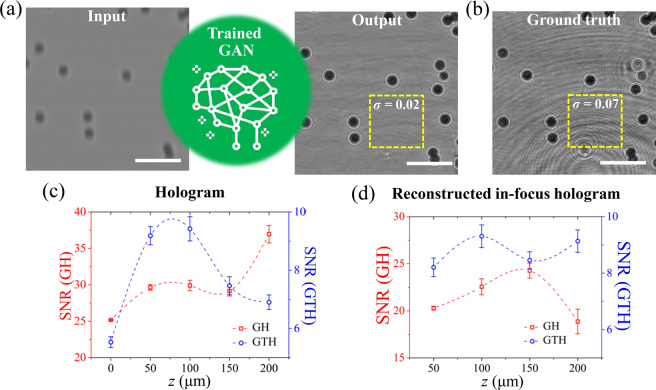
Performance of the deep learning-based image transformation. (**a**) GAN-based holographic image generated from a defocused BF image. (**b**) Ground truth holographic image of PS particles that correspond to the input BF image of PS particles. (**c**) Comparison of SNR values between the generated holographic image (output, GH) and the ground truth holographic image (GTH) as a function of the depth location (*z*) of particles. (**d**) Comparison of SNR values between the in-focus holograms reconstructed by the GHs and GTHs according to the depth location (*z*) of particles. Scale bars are 100 μm.

**Figure 4:**
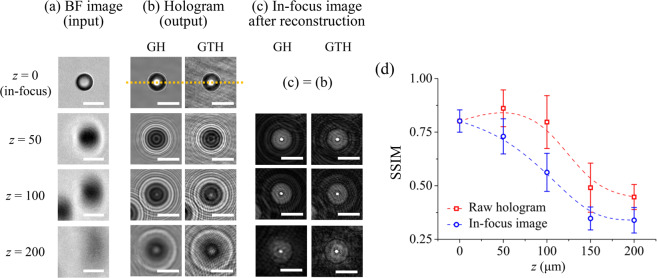
Comparison of particle holograms generated by the GAN and ground truth holograms as a function of depthwise location (*z*). BF images (input) of particles (**a**), generated (GH) and ground truth hologram (GTH) images of particles (**b**) positioned at three different particle depths (*z*) were compared. (**c**) Comparison of focused particle images from the generated and ground truth hologram images after numerical reconstruction. (**d**) Variations of the SSIM indexes of the raw holograms and focused images at various depth locations (*z*). An orange dotted line indicates the line passing through the center of each sample. Scale bars are 20 μm.

The performance of the trained GAN was evaluated by checking the image transformation results of PS particles (BF images → holographic images). BF and holographic images have intrinsic differences. Conventional optical microscopy with an incoherent light source has a DOF. DOF depends on the magnification of an objective lens. Typically, DOF is very short and usually measured in units of microns. Therefore, the BF images of PS particles located out of DOF became defocused and blurred [Fig. 3a]. The light signal of PS particles in a BF image weakened, and image blurring increased with the depth position of particles (*z*) [Fig. 4a]. Unlike conventional BF microscopy, DHM can record particle signals as interference patterns with a large observable depth by using a coherent laser beam [Fig. 3b]. The concentric interference fringes were generated for PS spherical particles [Figs. 3b and 4b]. The interference fringe spacing increased with the depth of the PS particle [Fig. 4b]. The degree of defocusing in BF images is closely associated with the interference patterns in holographic images. In the training stage, the GAN statistically learned this relationship by using several training image pairs (10^4^ for PS particles, 10^3^ for erythrocytes) to effectively convert BF images into holographic images.

Figure 3a shows a schematic of the proposed deep learning-based image conversion approach. After the training stage, real-like fake holographic PS particle images were generated from arbitrary defocused BF images by using the trained GAN [Fig. 3a]. The GAN was tested for samples that had no overlapping with the training sets. Figures 3a,b show the full field of views (FOV, 600 × 600 pixels) of typical generated holographic image and its ground truth image, respectively. Compared with the ground truth holographic image in Fig. 3, the background noise was largely reduced in the generated image. This kind of noise frequently appears in coherent imaging techniques, including DHM. The quality of hologram images can be degraded by such background noise. Noises can be induced by many resources, such as dust particles on the beam path, imperfections of various surfaces, multiple reflections from optical components, or misalignment of optical elements, etc. Thus, unexpected noises are formed in interference fringe patterns and speckle grains^35^. To quantitatively examine the background noise, the noise level was defined as the standard deviation (S.D., *σ*) of intensities of background, except for the particle signal. The background noise level *σ* was diminished from 5.0–7.0 × 10^−2^ in the ground truth to 1.0–1.6 × 10^−2^ in the generated hologram. The signal-to-noise ratios (SNRs) of the generated and ground truth holograms were also compared. In the present study, SNR is defined as follows^45^:

1 $$SNR=\frac{{\bar{I}}_{signal}}{\sigma }$$

where ${\overline{I}}_{signal}$ is the mean light intensity of holographic signals of particles and *σ* is the background noise level. Figure 3c shows variations of SNR values of generated hologram and ground truth hologram according to depth location (*z*) of particles. The range of SNR values of ground truth hologram was 5 to 10 [blue circles in Fig. 3c]. In contrast, the SNR values of generated hologram showed 3–5 times higher than those of ground truth hologram [red squares in Fig. 3c]. Since input BF images can not capture the signals induced by any imperfection out of DOF, fringe artifacts caused by any elements were not appeared in the holographic converted by trained GAN [Fig. 3a]. As a result, the background noise level *σ* and SNR were significantly improved in the generated holographic image. The improvement of SNR in the generated holograms gave rise to the increase of SNR of the reconstructed in-focus holograms from the generated holograms [Fig. 3d]. This noise reduction is especially important for the holographic imaging technique because various unwanted particles and features form interference fringes on the image plane and superimpose on the hologram of the sample [Fig. 4b] and degrade reconstructed image quality [Fig. 4c].

The generated and ground truth holograms of single particle at various depths were also compared to evaluate the generated hologram quality in more detail [Fig. 4]. As shown in Fig. 4b, the background noises of generated holograms were decreased, compared with those of ground truth holograms in all depths (*z* = 0–200 μm). However, the signal of input BF image became weak and unclear when the *z* positions of the particles were increased. Therefore, fine and high-order interference fringes, which are closely related to the quality of the hologram and the detailed 3D information of the particle, were not perfectly generated as *z* was increased. As a result, distorted hologram of particle was generated when *z* = 200 μm [Fig. 4b]. The numerically reconstructed holograms were also investigated [Fig. 4c and Supplementary Fig. S1]. The numerical reconstruction of the 2D holograms provides the in-focus images of particles in the full sample volume. The overall trends of in-focused images were the same with the raw hologram results Compared with the in-focus particle images reconstructed from the ground truth holograms, the in-focus images reconstructed from the generated holograms showed reduced background noise and increased SNR values [Fig. 3d and Supplementary Fig. S1]. Although SNR values were increased, the in-focus images reconstructed from the generated holograms exhibited less sharp boundaries of particles, compared to the in-focus images reconstructed from the ground truth holograms due to loss of high-order interference fringes in the generated holograms [Fig. 4c and Supplementary Fig. S1]. The structural similarity (SSIM) values of generated holograms (GAN output images) against the ground truth holograms with respect to depth (*z*) were compared in order to quantitatively evaluate the performance of the trained GAN. SSIM index is defined as follows:

2 $$SSIM({I}_{GH},{I}_{GTH})=\frac{(2{\bar{I}}_{GH}{\bar{I}}_{GTH}+{C}_{1})(2{\sigma }_{GH,GTH}+{C}_{2})}{({\bar{I}}_{GH}^{2}+{\bar{I}}_{GTH}^{2}+{C}_{1})({\sigma }_{GH}^{2}+{\sigma }_{GTH}^{2}+{C}_{2})}$$

where ${\overline{I}}_{GH}$ and ${\overline{I}}_{GTH}$ are the mean intensity values of the images *I*_*GH*_ and *I*_*GTH*_, respectively. *σ*_*GH*_ and *σ*_*GTH*_ are the standard deviation of *I*_*GH*_ and *I*_*GTH*_, respectively and *σ*_*GH,GTH*_ is the covariance between the two images. *C*_1_ and *C*_2_ are constants used to prevent division by a small denominator. Based on these definitions, Fig. 4d shows the variations of SSIM values of raw holograms and in-focus images as a function of depth (*z*). As a result, SSIM values of raw holograms and in-focus images decreased with increasing depths (*z*). When particle depth position is greater than 150 μm, SSIM values are less than 0.5. These SSIM results also reflect the imperfect hologram generations of the particles located at deep depths.

### 3D positioning using generated holograms

A single particle hologram encrypts the 3D volumetric field information. An important feature extracted from the hologram is the 3D positional information of individual particles from the amplitude image of the numerically reconstructed image. We can acquire the 3D positional information of individual particles and evaluate how precise this information can be obtained from the holograms converted from BF images by the trained GAN.

**Figure 5:**
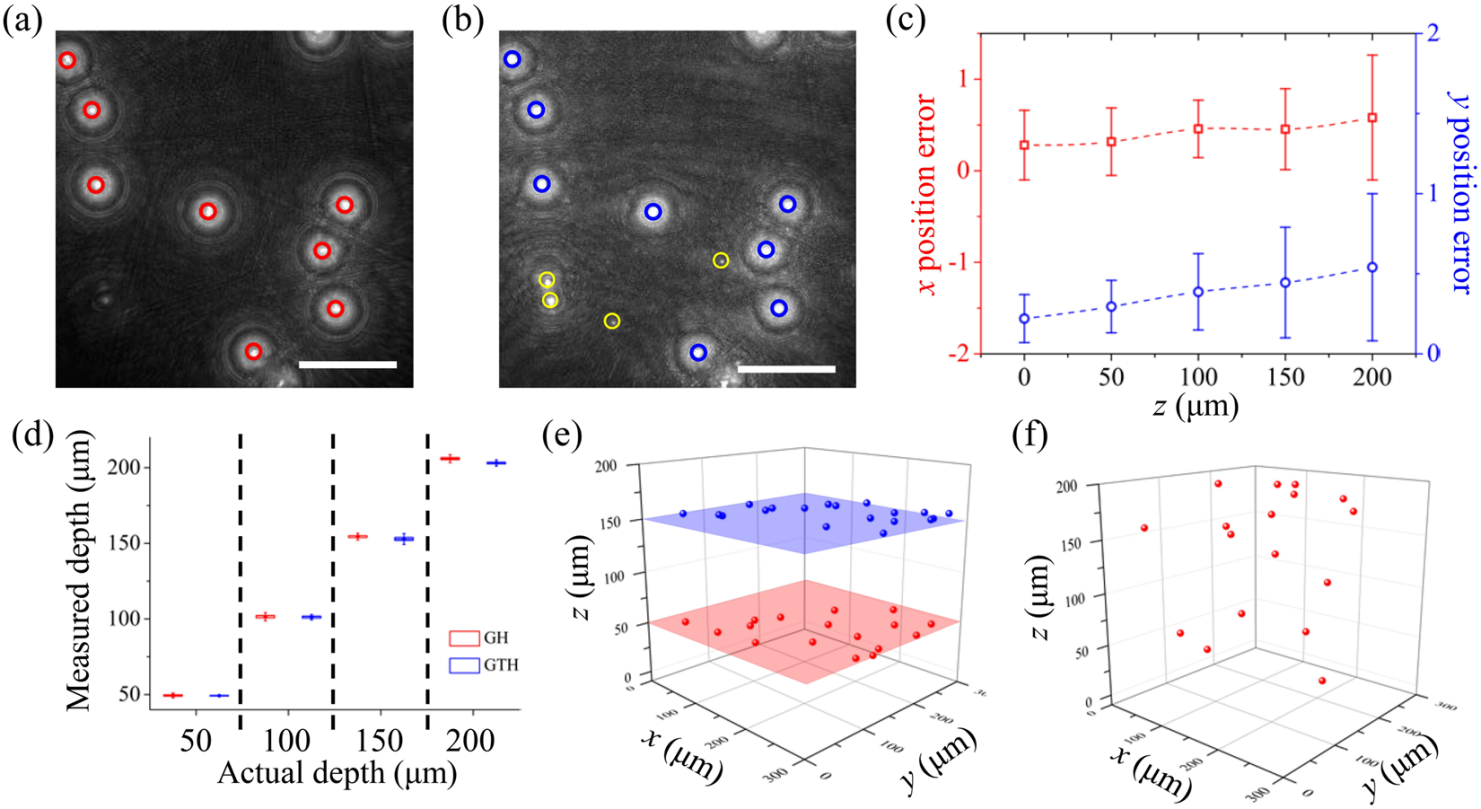
Acquisition of 3D positional information of microparticles. Projection images of the reconstructed particle images from (**a**) the generated holographic image (GH) and (**b**) the ground truth holographic image (GTH). (**c**) Variations in coplanar (*x*, *y*) position errors as a function of particle depth location (*z*). (**d**) Comparison of the depth positions (*z*) obtained from the generated and the ground truth holograms. (**e**) Spatial distributions of particles at two different *z* planes. (**f**) Spatial distribution of particles arbitrarily dispersed in a 3D volume. Scale bars are 100 μm.

To determine the in-plane (*x*, *y*) positions of particles, the numerically reconstructed images were projected onto a 2D single plane after a holographic image was numerically reconstructed [Fig. 5a,b]. In the projection image, the local intensity peaks appeared at the centers of the microparticles. Therefore, the in-plane (*x*, *y*) positions of particles were determined by locating the points of local maximum intensities in the projection image. Figures 5a,b show the projection images of the reconstructed particle images from a generated holographic image and the ground truth holographic image, respectively. The local intensity peaks of particles (blue circles) and unexpected signals (yellow circles) in the ground truth hologram are shown simultaneously in Fig. 5b. However, only particle signals (red circles) were detected in the projection image of the generated hologram due to the increase of SNR values [Fig. 5a]. The errors in the in-plane (x, y) position measurement from the real in-plane positions extracted from the ground truth hologram were analyzed. Figure 5c and Table 1 show the error variations according to the depth location (*z*) of particles. As the SSIM values decreased with the increase in particle location *z* [Fig. 4d], the in-plane position (x, y) errors and the corresponding RMS errors also gradually increased [Fig. 5c and Table 1]. The maximum RMS position error within this range of particle depth (0–200 μm) is 0.89 μm. Considering the spatial resolution in the image plane (20×, 0.53 μm/pixel), the accuracy of in-plane positioning from the generated hologram is sufficiently high.

**Table 1:** Measurement errors of in-plane (*x*, *y*) positions according to the depth location (*z*) of particles.

	*x*_error_ (μm)		*y*_error_ (μm)		
	Mean ± S.D.	RMS error	Mean ± S.D.	RMS error	
Depth (μm)	0	0.28 ± 0.38	0.41	0.22 ± 0.15	0.27
	50	0.32 ± 0.37	0.47	0.30 ± 0.16	0.33
	100	0.46 ± 0.31	0.55	0.39 ± 0.24	0.41
	150	0.45 ± 0.44	0.62	0.44 ± 0.35	0.55
	200	0.58 ± 0.68	0.89	0.54 ± 0.46	0.70

After extracting the in-plane positions of individual particles, segmented images were generated around the in-plane positions of each particle. The segmentation process was conducted for all the reconstructed images along the depth direction. The depth (*z*) positions of microparticles were determined by applying an autofocus function to the segmented images^19,46,47^. Autofocus functions quantify the degree of image sharpness (i.e., focus value) in the segmented area by scanning a series of segmented reconstructed images of each particle. As the autofocus function, we adopted the 2D image variance (*VAR*), which is defined as follows:

3 $$VAR(z)=\frac{1}{{N}_{x}{N}_{y}}\sum _{x,y}{\{I(x,y,z)-\bar{I}(z)\}}^{2},$$

where *I* and $\overline{I}$ denote the intensity distribution and its average, respectively. *N*_*x*_ and *N*_*x*_ are the image pixel dimensions. The reconstruction depth, which has the maximum focus value, was determined as the actual *z* position of each particle [Supplementary Fig. S2]. The performance of the depth positioning in the generated holograms was evaluated by using a planar test target. 5 μL of a diluted particle suspension was placed between two flat plates. The planar test target comprises a bottom slide glass (26 × 76 × 1 mm^3^) and a top cover slip (24 × 24 × 0.15 mm^3^). Microparticles dispersed in the solution nearly constituted a monolayer. The test target was precisely positioned at four different depths (e.g., 50, 100, 150, and 200 μm) away from the focal plane by adjusting a *z*-axis translation stage with 1 μm precision. The uncertainty in the measurement of depth positions was examined by using a planar target^19,22^.

Figure 5e shows the spatial distributions of particles located between two planar glasses positioned at two different *z* planes. The particles are clearly positioned at the targeted locations. Table 2 summarizes the measured depth positions and their RMS errors acquired from the generated and ground truth holograms. Although the maximum image sharpness (focus value) of the generated holograms was smaller than that of the ground truth holograms, the peak reconstruction depth positions (i.e., particle depth positions) were maintained [Supplementary Fig. S2]. The S.D. in the measured depth positions, the corresponding RMS errors obtained from the generated holograms, and the difference in RMS errors between the generated hologram and ground truth hologram gradually increased with the depth *z* [Fig. 5d and Table 2], because high-order interference patterns were lost in the generated holograms and SSIM values steadily decreased with the increase of depth *z* [Fig. 4b,d]. Although the performance of depth position measurements using the generated holograms was poorer than that using the ground truth holograms, the depth locations (*z*) acquired from the generated holograms matched well with the actual target depth positions [Fig. 5d,e, and Table 2]. The spatial distribution of particles arbitrarily dispersed in a 3D volume was also acquired from the holograms converted from BF images of particles [Fig. 5f and Supplementary Fig. S3].

**Table 2:** Comparison of the depth positions and their RMS errors obtained from the generated and ground truth holograms of particles.

		*z*_GH_ (μm)		*z*_GTH_ (μm)	
		Mean ± S.D.	RMS error	Mean ± S.D.	RMS error
Actual depth (μm)	50	49.39 ± 1.59	1.59	49.23 ± 0.59	0.95
	100	101.47 ± 2.68	2.95	101.11 ± 1.75	2.00
	150	154.46 ± 2.22	4.94	152.91 ± 3.62	2.93
	200	206.00 ± 2.69	6.53	203.11 ± 2.07	3.70

### Light scattering pattern acquisition from generated hologram

Light scattering characteristics can also be extracted from the captured holograms. The light scattering patterns contain the morphological and optical features, such as shape, size, refractive index distributions, and orientation with respect to the incident beam^6,7,48–51^. When a planar monochromatic beam propagates through a sample, the beam is refracted at the edges of the sample, because the sample behaves as an optical microlens. The beam is diverged or focused at certain spots. The locations of the focused points depend on the morphology and optical properties of the sample. Therefore, light scattering patterns can be used as a valuable feature to characterize individual samples.

**Figure 6:**
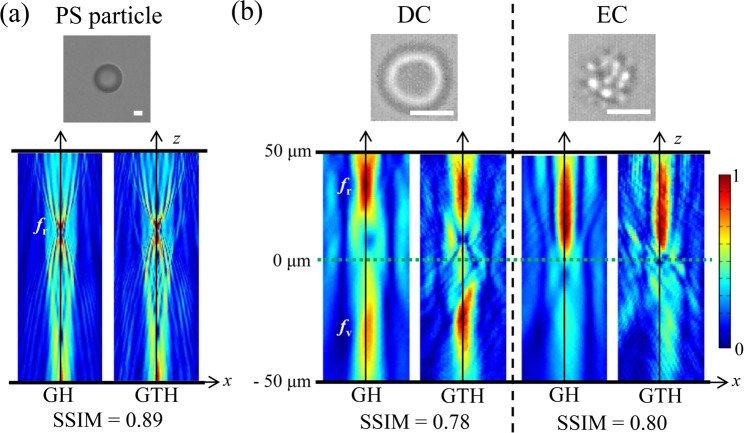
Acquisition of light scattering patterns of microparticles. Light scattering patterns of (**a**) a PS particle and; (**b**) erythrocytes (DC: discocyte, EC: echinocyte) derived from the generated holograms (GHs) and the ground truth holograms (GTHs). *f*_r_ and *f*_v_ represent the real and virtual foci, respectively. Scale bars are 5 μm.

The light scattering patterns were derived from the numerically reconstructed images of test samples at various depths (*z*). The intensity profiles along a line that passed through the center of each reconstructed sample image [orange dotted line in Fig. 4b] were stacked along the *z*-axis. Finally, the light intensity distribution maps of the samples in the *x*-*z* plane were obtained [Fig. 6].

The light scattering patterns of PS particle and erythrocytes were acquired from the generated and ground truth holograms and compared in Fig. 6. For PS particles, strong intensity peaks appeared at real foci (*f*_*r*_) in both light scattering patterns because the PS particle functioned as a spherical lens [Fig. 6a]. The light scattering patterns obtained from the generated hologram were almost similar to those obtained from the ground truth hologram; the SSIM value was 0.89.

The light scattering patterns of erythrocytes acquired from the generated and ground truth holograms are shown in Fig. 6b. The prolonged period of blood storage has altered the structural morphology of erythrocytes. During the 14-day storage of erythrocytes, two types of erythrocytes, namely discocyte (DC) and echinocyte (EC), were investigated. In the light scattering patterns of DC acquired from the generated and ground truth holograms, two bright spots are appeared at the real focus (*f*_*r*_) and virtual focus (*f*_*v*_) [Fig. 6b]. Virtual focus was generated because of the central dimpled shape of DC. Real focus was created because of the toroidal shape at its edge. Although the light intensity at the virtual focus acquired from the generated hologram was slightly decreased, the location of virtual focus was almost similar to that acquired from the generated hologram. As the preservation period increases, DC undergoes morphological change to EC. EC has an irregular shape with bumpy and spicular structures on its surface. As DC is changed to EC, its concavity at the center disappears with the increase in overall sphericity. As a result, a bright spot is formed only at the real focus in the light scattering pattern of EC [Fig. 6b]. The real focus of EC is shown in all light scattering patterns acquired from the two holograms. The SSIM values for erythrocytes are above 0.75. Although the SSIM values for erythrocytes are not as high as the value for PS particle, it has little effect on the extraction of important features for characterizing erythrocytes in the present study. The real focal lengths acquired from the generated holograms are 36.5 ± 5.7, 18.3 ± 4.9 μm for DC (n = 10), and EC (n = 10). The virtual focal length (*f*_v,_) of DC (n = 10) is 28.9 ± 4.8 μm. These values are in good agreement with our previous result^6^.

The present GAN-based image conversion technique would extend the application of conventional BF (i.e., optical) microscopy to various fields, such as the analysis of particle dynamics and detection of abnormal cells. Several information of the hologram transformed from a BF image, which cannot be obtained from original BF microscopy, can be acquired. For example, the 3D positional information of particles and spatial distribution of particles in a 3D volume can be extracted from the converted hologram [Figs. 5e,f]. If the 3D positions of individual samples were tracked as a function of time by applying a particle tracking velocimetry (PTV) algorithm, the 3D velocity field or 3D trajectories of test samples can also be acquired without the need of depthwise scanning procedures^18,22,24,25,42^. The light scattering patterns extracted from the converted hologram make the precise prediction of the 3D positions of transparent microspheres and the orientations of transparent non-spherical particles^48,49^ as well as the accurate diagnosis of erythrocyte alterations due to long period of blood storage for transfusion and malaria with more than 95% precision, possible^6,7,50,51^. Although we extracted 3D positional information and light scattering pattern from the amplitude images of the numerically reconstructed images in this study, valuable biophysical features can be additionally extracted from the phase image acquired by training off-axis holograms or adopting phase retrieval algorithms^21,52,53^. In addition, a coherent or partially incoherent light source and additional optical components are not necessary to generate holograms of particles, if the trained GAN is utilized. Therefore, the experimental setup for conventional DHM can be more inexpensive and simplified. In general, the trained network can generate holograms from which 3D particle distribution can be extracted [Fig. 3a and Supplementary Fig. S3]. However, the network might be difficult to generate holographic images effectively, when particles are overlapped too much [Supplementary Fig. S5]. The data diversity in the training data set may solve this problem by improving generalization ability and performance of the deep learning network.

We proposed and experimentally validated a GAN-based framework that can convert BF images into holographic ones and its usefulness. The GAN was trained with numerous BF and holographic image pairs acquired using the hybrid imaging system incorporated with a simple microfluidic device. It also learned the statistical image transformation. The performance of the developed GAN-based framework was demonstrated for PS particles and biological erythrocyte samples. The image conversion results were quantified using SNR and SSIM values. As a result, the hologram converted from a BF image using the trained GAN successfully provided the 3D positional information and light scattering pattern. The proposed GAN-based methodology has two major advantages of (1) extracting valuable features from BF images for identification of particles in a volume and (2) creating holograms with a conventional BF imaging setting. Therefore, the technique could be applied to analyze the 3D dynamics of particles or cells and accurately detect or diagnose abnormal cells by using a general white light source.

## Methods

### Sample preparation

Transparent spherical polystyrene (PS) microparticles with an average diameter of 14.92 ± 0.03 μm were tested. The density of the polystyrene particle is *ρ* = 1.05 g/mL. The particles were mixed with NaCl solution, which had the same density as the PS particle. The volume fraction of PS particles was 1%. A 20× water immersion objective lens was utilized to record the magnified BF and hologram images of the PS particles.

Blood was extracted from male Sprague–Dawley rats (11 weeks old, 350–400 g) through abdominal aortic puncture. The blood was initially collected in an ethylenediaminetetraacetic acid (EDTA) vacuum tube (Vacutainer K3 EDTA, BD, USA). Then, citrate phosphate dextrose adenine-1 (CPDA-1, C4431, Sigma-Aldrich, USA) was added to the EDTA-treated blood at a 1:7 v/v ratio to prevent blood coagulation and variations in biophysical properties. The resultant mixture was stored in the dart at 4 °C for 2 weeks. For each experiment, 3 µL of blood was collected from the blood solution. The collected blood sample was diluted with phosphate buffer saline (PBS) solution to a hematocrit of 1%. The condition of low volume fraction (1%) was tested in this study to minimize the unexpected noise caused by particle-to-particle interferences. A 60× water immersion objective lens was used to capture the magnified BF and hologram images of erythrocytes. All experimental protocols and procedures were approved by the Animal Care and Ethics Committee of Pohang University of Science and Technology (POSTECH), and all experiments were conducted in accordance with the approved guidelines (POSTECH-2019–0026). Each experiment was carried out at 25 °C within 30 min.

Suspensions of PS particles and erythrocytes were infused into a 100-mm long transparent rectangular microchannel (width: 500 µm, height: 50 µm, VictroCom, USA) to gather considerable number of BF-hologram image pairs that will be used for the training network [Fig. 1a]. The channel was made of borosilicate glass. The Reynolds number was maintained at 10^−2^ by adjusting the flow rate (approximately 0.2 µl/min). The focal plane of the objective lens was adjusted using the *z*-axis translation stage of a microscope. Finally, numerous BF images and corresponding holographic microparticle images located at various depth positions (*z*) were captured by using this simple microfluidic device.

### Hybrid bright-field and holographic imaging system

The hybrid imaging system was used to simultaneously record BF images and the corresponding holographic particle images^42^. Figure 1a shows the experimental setup of the hybrid BF-hologram imaging system. A halogen lamp (white beam) and a continuous Nd:Yag laser (*λ* = 532 nm, 60 mW, Crystal Laser, USA) were used as the light sources for BF and hologram imaging, respectively. The laser beam was spatially filtered and collimated. The white and collimated laser beams illuminated the microparticles that flowed in the rectangular microchannel. Water immersion objective lenses (20×, 60×, Nikon, Japan) magnified the BF and holographic images of spherical particles and erythrocytes. The tube lens inside a microscope (Eclipse 50i, Nikon, Japan) made the beam less divergent. A dichroic mirror was located behind the tube lens. The mirror only reflected green light and passed all other lights. As a result, the beam was split into two beams for BF and hologram imaging. Only BF images were recorded using the first high-speed CCD camera (pco. 1200 hs, PCO, Germany) [Fig. 1b]. BF images were consecutively acquired at a frame rate of 10 fps with an exposure time of 1 ms, which correspond to the minimal value available for the present optical setup to clearly record the microparticle holograms without the blurring effect caused by particle motion. Holographic images were captured by using second high-speed CCD camera (FASTCAM Mini UX100, Photron, Japan) at the same frame rate used for recording BF images with an exposure time of 25 μs [Fig. 1c]. These two CCD cameras were connected to a delay generator to trig their exposures for obtaining BF and corresponding hologram images at the same time. The pixel resolution of the CCD cameras was 1280 × 1024 pixels. The spatial resolutions in the image plane of both imaging techniques were 0.53 μm/pixel (20×) and 0.18 μm/pixel (60×). Linear translation stages installed with a micrometer were attached to both of the CCD cameras to carefully adjust the distance between the tube lens and each CCD camera. These CCD cameras were located 215 mm away from the tube lens.

### GAN for converting BF image to holographic image

A GAN was implemented to convert a BF image into a holographic image in the present study. The adversarial training of GAN architectures are reported to generate more realistic ouput data, compared to the architecture without adversarial training^40,43,44^. The GAN is composed of a generator and discriminator network as shown in Fig. 2a. In the generator, a variation network of the original multiscale CNNs^43^ was used to transform BF images into holographic images. Input images (I_0_, I_1_, I_2_, I_3_) with different pixel resolutions are provided to the generator, by interpolating the input BF image (I_0_) having the largest pixel size (n_x_ × n_y_) to images (I_1_, I_2_, I_3_) with smaller pixel resolutions (n_x_ × n_y_/2^2^, n_x_ × n_y_/2^4^, n_x_ × n_y_/2^8^). By providing the input images with different pixel resolutions, the multiscale CNNs can learn structures containing multirange spatial dependencies^44^. Therefore, the multiscale CNNs are expected to be capable for learning mappings that contain multirange spatial dependencies such as interference patterns of particles. A variation structure of a discriminator proposed by the previous research^31^ was used for the discriminator of the present GAN. The discriminator extracted features of holographic images using CNNs and classified them by giving scores between 0 to 1 in fully connected layers, where the values close to 0 and 1 indicate generated holograms and ground truth holograms, respectively.

Let *G*(*I*) and *GT*(*I*) be the generated and ground truth holograms from BF images (*I*), and let *D*(*G*(*I*)) and *D*(*GT*(*I*)) be the corresponding scores from the discriminator for the hologram images. Then, the generator loss is calculated as follows:

4 $${L}_{G}=MSE(G(I),\,GT(I))+BCE(D(G(I)),1)$$

and the discriminator loss is expressed as follows:

5 $${L}_{D}=BCE(D(G(I)),\text{0})+BCE(D(GT(I)),\text{1})$$

where *MSE* and *BCE* correspond to the mean squared error and binary cross-entropy loss functions, which are calculated as follows:

6 $$MSE(x,y)=\sqrt{\mathop{\sum }\limits_{i,j}^{{n}_{x},{n}_{y}}({y}_{i,j}-{x}_{i,j})/({n}_{x}\times {n}_{y})}$$

7 $$BCE(x,y)=-\,y\,\log (x)-(1-y)\log (1-x)$$

The generator was trained with an Adam optimizer^54^ to minimize generator loss with a learning rate of 4 × 10^−6^. Furthermore, the discriminator was trained with a stochastic gradient descent method to minimize discriminator loss with a learning rate of 2 × 10^−2^, momentum of 9 × 10^−1^, and weight decay of 5 × 10^−5^. The network was trained and tested by using a PC with a Nvidia GTX 1070 Ti GPU, a Core i7–5960K CPU running at 3.0 GHz, and 32 GB of RAM. The network was programmed by using Python (version 3.6.0) with the Pytorch library (version 1.0).

The network’s training dataset was made up of images of PS particles and erythrocytes that flow in a microchannel. A total of 10,000 image pairs of BF and holographic images of PS particles at depths (*z*) ranging from 0 μm to 200 μm were used as training datasets. In addition, 1,050 image pairs of blood samples were also utilized for training. The erythrocytes tested in this study were located at 0 < z < 50. All the obtained image pairs show different particle distributions without overlapping one another. The pixel resolution of all images used for training was 800 × 800 pixels. Four pairs of BF and holographic images, which corresponds to the number of batches with 256 × 256 pixels, were randomly cropped from a random selection of image pairs from the training data sets in each iteration of training. During testing, images with a large size can be generated because the discriminator network that contains fully-connected layers is not used during testing. Test data sets consisted of 2,000 and 150 image pairs for PS particles and erythrocytes, respectively. The utilized GAN was trained up to 500,000 iterations. *L*_2_ errors of test data, which are the square root of *MSE*, converged as the training iteration increased [Fig. 2b]. After the training stage, realistic fake holographic images were generated from arbitrary BF images by using the trained GAN. Compared to the multiscale CNNs, the GAN produced holograms more similar to the ground truth holograms [Supplementary Fig. S4].

### Hologram reconstruction

Diffraction occurs when a coherent laser beam irradiates a microparticle located in the pathway of the beam. The interference between the diffracted object wave and unaffected reference wave creates a microparticle hologram in the image plane [Fig. 1c].

Afterwards, the holographic images were numerically reconstructed by adopting the angular spectrum method along the depth direction (*z*)^55^. The complex wavefield at any plane perpendicular to the optical axis (*z*) can be expressed as follows:

8 $$\varGamma (\xi ,\eta ,z)={F}^{-1}\left[F\{h(x,y)\}\exp (iz\frac{2\pi }{\lambda }\sqrt{1-{(\lambda {f}_{\xi })}^{2}-{(\lambda {f}_{\eta })}^{2}})\right]$$

where *F* and *F*^−1^ denote the fast Fourier transform and its inverse, respectively. (*x, y*) and (*ξ, η*) correspond to the spatial coordinates in the holographic and reconstructed image planes, respectively. *f*_*ξ*_ and *f*_*η*_ represent the corresponding spectral coordinates. Function *h* (*x*, *y*) represents the hologram at the image plane, *z* is the distance between the hologram and reconstruction planes, and *λ* is the wavelength of the laser beam. The convolution magnitude $|\varGamma (\xi ,\eta ,z)|$ was evaluated to obtain an amplitude image of the reconstructed image. A total of 301 reconstructed images were generated from a single holographic image because the reconstruction volume covered from 0 μm to 300 μm along the *z* direction with intervals of 1 μm.

## Supplementary information

Supplementary information.
