# Supplementary material for: Deep learning-based hologram generation using a white light source
Source: Sci Rep. 2020 Jun 2;10:8977. doi: 10.1038/s41598-020-65716-4 (PMC7265409; doi:10.1038/s41598-020-65716-4)
Supplement: Supplementary file 1 — Supplementary information. [file 41598_2020_65716_MOESM1_ESM.pdf]

Supplementary Information

Deep learning-based hologram generation using a white light source

Taesik Go[a], Sangseung Lee[b], Donghyun You[b], Sang Joon Lee[a*]

[a] Center for Biofluid and Biomimic Research, Department of Mechanical Engineering, Pohang University of Science and Technology, Pohang, 37673, Republic of Korea

[b] Flow Physics and Engineering Laboratory, Department of Mechanical Engineering, Pohang University of Science and Technology, Pohang, 37673, Republic of Korea

*Corresponding Author: **Prof. Sang Joon Lee**

Center for Biofluid and Biomimic Research, Department of Mechanical Engineering, Pohang University of Science and Technology, Pohang, 37673, Republic of Korea

E-mail: sjlee@postech.ac.kr

Phone: +82-54-279-2169, Fax: +82-54-279-3199

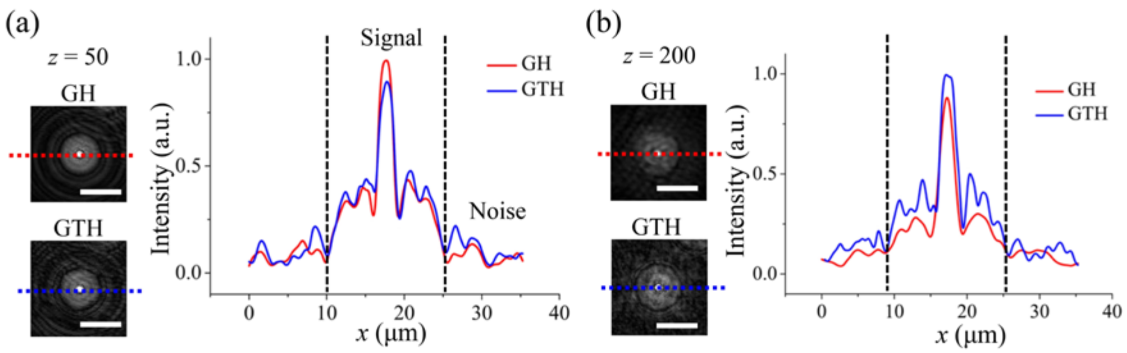

Supplementary Figure S1. Intensity profiles along the horizontal lines passing through the centers of the in-focus holograms reconstructed from the generated (GH) and ground truth hologram (GTH) images of particles located at two different depth positions (z = 50 and 200 μm). Scale bars are 20 μm.

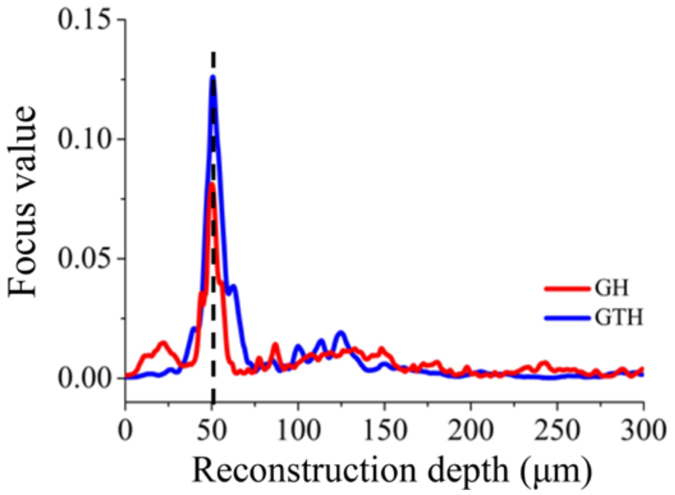

Supplementary Figure S2. Variations of the focus value along the reconstruction depth (z) acquired from the generated and ground truth holograms.

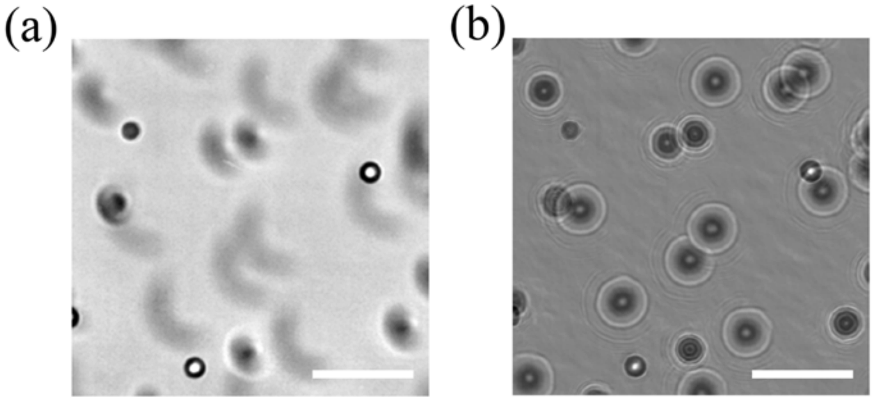

Supplementary Figure S3. (a) Bright-field images and (b) generated holograms of particles located at different depth locations (z). Scale bars are 100 μm.

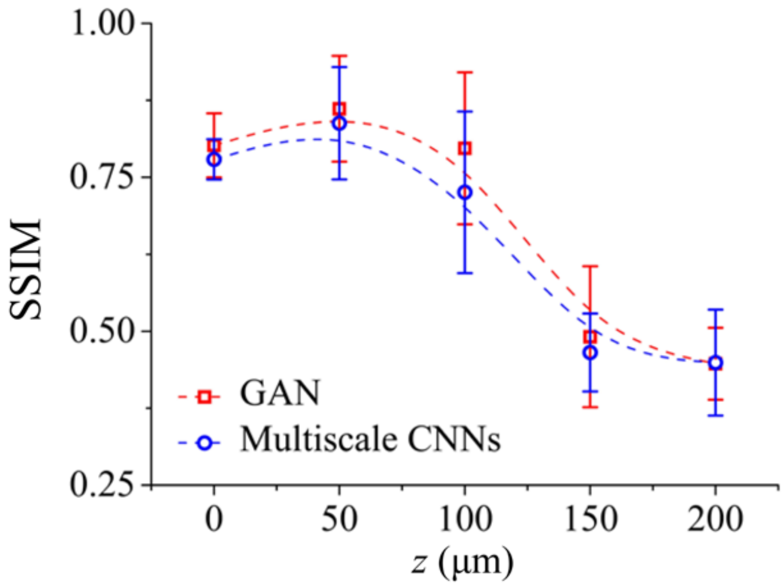

Supplementary Figure S4. Comparision of the SSIM indexes of the holograms generated by the GAN and the multiscale CNNs against the ground truth holograms.

(a) (b) (c)

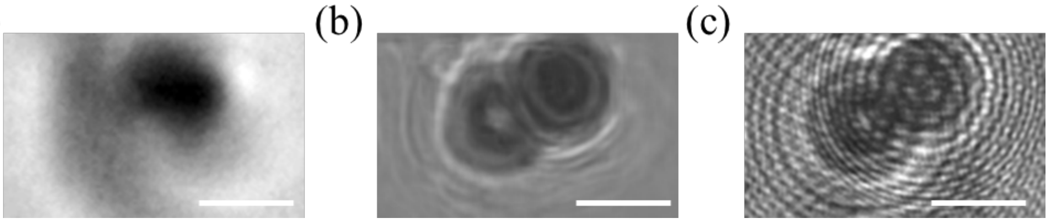

Supplementary Figure S5. Failure in the generation of holograms of overlapped particles. (a) Input bright-field image, (b) generated holograms and (c) ground truth holograms of overlapped particles. Scale bars are 20 μm.
